# Factors that may impact on immunosenescence: an appraisal

**DOI:** 10.1186/1742-4933-7-7

**Published:** 2010-06-14

**Authors:** Joseph Ongrádi, Valéria Kövesdi

**Affiliations:** 1 Institute of Public Health, Semmelweis University, Budapest, Hungary

## Abstract

The increasing ratio of ageing population poses new challenges to healthcare systems. The elderly frequently suffer from severe infections. Vaccination could protect them against several infectious diseases, but it can be effective only if cells that are capable of responding are still present in the repertoire. Recent vaccination strategies in the elderly might achieve low effectiveness due to age-related immune impairment. Immunosenescence affects both the innate and adaptive immunity.

Beside individual variations of genetic predisposition, epigenetic changes over the full course of human life exert immunomodulating effects. Disturbances in macrophage-derived cytokine release and reduction of the natural killer cell mediated cytotoxicity lead to increased frequency of infections. Ageing dampens the ability of B cells to produce antibodies against novel antigens. Exhausted memory B lymphocyte subsets replace naïve cells. Decline of cell-mediated immunity is the consequence of multiple changes, including thymic atrophy, reduced output of new T lymphocytes, accumulation of anergic memory cells, and deficiencies in cytokines production. Persistent viral and parasitic infections contribute to the loss of immunosurveillance and premature exhaustion of T cells. Reduced telomerase activity and Toll-like receptor expression can be improved by chemotherapy. Reversion of thymic atrophy could be achieved by thymus transplantation, depletion of accumulated dysfunctional naive T cells and herpesvirus-specific exhausted memory cells. Administration of interleukin (IL)-2, IL-7, IL-10, keratinocyte growth factor, thymic stromal lymphopoietin, as well as leptin and growth hormone boost thymopoiesis. In animals, several strategies have been explored to produce superior vaccines. Among them, virosomal vaccines containing polypeptide antigens or DNA plasmids as well as new adjuvanted vaccine formulations elicit higher dendritic cell activity and more effective serologic than conventional vaccines responses in the elderly. Hopefully, at least some of these approaches can be translated to human medicine in a not too far future.

## 1. Background

The global population, especially in the developed countries, is ageing. The percentage of the population that is elderly (≥60 years of age) now represents a larger proportion than ever before: it has increased from 8% in year 1950 to 10% in 2000, and this trend is expected to continue, to reach 21% of the population by 2050 [1]. People are living much longer than they used to and the longer they live, the longer their bodies are exposed to environmental factors which increase the risk of age-associated diseases [2]. The elderly suffer from more frequent and more severe community-acquired and nosocomial infections than younger people, and they tend to experience poor outcomes from infections in comparison to the younger population [3,4]. The clinical presentation is often atypical creating diagnostic difficulties. Latently carried intracellular pathogens such as viruses (e.g. members of *Herpesviridae)*, bacteria (e.g. Mycobacteria) or fungi (e.g. Candida) reactivate and opportunistic infections manifest themselves at increased rates [5]. In Western countries, the mortality rate increases in people over 65 years, if compared to individuals between 25- and 44-year old, e.g. 89-fold for pneumonia and influenza or 43-fold for cancer [2]. Collectively, these diseases severely influence the quality of life of the elderly and their families and greatly challenge public healthcare systems. Therefore, prevention of these infections becomes critically important. The most important reason for the increased rate of infections (and cancers) in the elderly is believed to be the diminished or exhausted function of the immune system which occurs with ageing (immunosenescence, immune exhaustion). Vaccination could protect the elderly against several infections and possibly cancer [1,4,6], but at least a partial restoration of age-related immune deficits seems to be a pre-requisite for the success of any vaccination regimen in older people.

## 2. Natural factors affecting the ageing of the immune system and their elimination

Immunosenescence due to deregulated immunity [7] is a very complex process and remains to be fully understood [1]. Normal ageing is determined genetically, but several external factors might affect immunosenescence positively or negatively. Indeed, according to modern views the actual state of the immune system in the elderly is the result of a continuous remodelling process [8]. Oxidative stress is believed to be a major factor of accelerated ageing, possibly due to an increased pace of telomere shortening resulting from DNA damage. Telomeres are DNA+protein complexes at the end of chromosomes and are supposed to be the molecular clock of ageing, including that of the immune system, especially lymphocytes [9]. The shortening of telomeres is due to diminished activity of telomerase that fails to add telomere repeat sequences to the end of chromosomes [2,9]. Senescence can be prevented or reversed using telomerase-based approaches [1]. Gene therapy using catalytic human telomerase could improve the function of immune cells [10]. Furthermore, chemotherapeutic agents acting on the catalytic component of human telomerase, such as TAT2, a small molecule telomerase activator, could stabilise telomerase length and retard loss of immune control over microbial infections [2]. Biotech companies ought to take the challenge of finding additional ways to prevent telomerase shortening.

Gender differences in life expectancy are partially based on altered immune functions: e.g. androgen hormones are known to contribute to thymic involution. Sociodemographic factors also exert a major impact on susceptibility to age-related diseases; these include residency, institutionalisation, income, level of education, life style and disability in daily living. Unhealthy habits, comorbidities and medications also contribute to declining immune activity. Among these, it is worth mentioning smoking, alcoholism, chronic obstructive pulmonary diseases, hypertension, stroke, heart failure, diabetes mellitus, rheumatic and autoimmune diseases and treatments with chronic oral glucocorticosteroids, as well as severe cognitive impairment, Alzheimer disease [2], chronic stress, chronic antigenic stress with consequent inflammation, and many others [11]. Malnutrition is associated with a decrease in immunity and an increase in susceptibility to many infectious diseases. Both of these effects are exacerbated by ageing. Underweight may contribute to an increased mortality by some infectious diseases due to an inability to meet the energy demands associated with the immune response to selected microbial infections [12]. Interventions on nutrition could have a larger impact on immune functions than commonly appreciated. Vitamin A contributes to the maintenance of epithelium integrity in the respiratory and gastrointestinal tracts. Vitamin E regulates lipid rafts and membrane fluidity on the surface of immune cells and reconstitutes immunological synapse formation, as clearly demonstrated in naive CD4^+ ^T cells of old mice [5,13]. Vitamin E supplementation reduces the risk of influenza virus infection in aged people. Vitamin D enhances activation of Toll-like receptors (TLRs) and increases cathelicide production, the latter of which contributes to the destruction of intracellular *Mycobacterium tuberculosis*. Among the trace elements, zinc is especially important because it maintains the activity of more than 300 enzymes including those in polymorphonuclear phagocytes, natural killer (NK) cells and the complement cascade [5]. Anti-inflammatory nutritional intervention could be especially useful [14]. Modulating lipid intake may also be beneficial; e.g. conjugated linoleic acid can result in decreased pro-inflammatory cytokine secretion and has been reported to increase the success rate of hepatitis B vaccination in the elderly [15]. The lipid environment strongly influences T cell functions through alterations in the membrane fluidity of these cells [16]. Human high density lipoprotein (HDL) has anti-inflammatory and anti-oxidative effects; HDL extracts accumulated cholesterol in lipid rafts, resulting in increased T cell receptor (TCR) signal transduction and T cell activation [17]. Caloric restriction, that was shown to improve immune responsiveness in rodents [18], has also been found to delay T lymphocyte immunosenescence in non-human primates, preserving the number and function of naive T cells, and to reduce pro-inflammatory states [19]. Moderate exercise also serves as an anti-ageing common-sense medical advice. Aerobic exercise, weight loss and cessation of smoking can raise HDL levels [17], while participation in regular physical activity has been seen to lower the increase in proinflammatory cytokine IL-6 and C-reactive protein (CRP) that occurs with age [8]. Elderly people with known risk factors could be advised to reduce exposure to these factors (e.g. to stop smoking or unnecessary use of glucocorticoids) [11]. Not only all types of malignancy but also many therapeutic procedures used in their treatment impair the immune functions in a vicious circle. Chemo- and radiotherapy, as well the immunosuppressive drugs used in connection with organ transplantation are well known examples [11].

Beside individual genetic predisposition to immunosenescence, epigenetic changes accumulating over the full course of human life in the cells due to environmental effects basically determine the quality of life in the elderly. Major epigenetic mechanisms include DNA methylation, histone modifications such as methylation, acetylation, phosphorylation, structural modifications of the chromatin, and the preferential production of selected microRNAs. DNA hypomethylation in CpG rich, promoter-associated regions and acetylated histones allow active transcription, while DNA hypermethylation and histone hypoacetylation promote gene silencing. The changes may be one or two orders of magnitude greater than the rate of somatic mutations. They are reproduced during DNA replication and are therefore stably transmitted to the daughter cells including immune cells. Diet in particular can influence methylation by modulating the availability of methyl donors including folate, choline, and methionine. Low levels of methionine early in life results in longer life and relative preservation of immune functions. This supports an important role of methyl diet and life-extending caloric restriction. Protein restricted diet of rats induced hypomethylation. Lymphocyte function-associated antigen-1 (LFA-1), which is involved in T cell activation, is overexpressed in the T cells of elder people and patients with systemic lupus erythematosus (SLE). Inhibition of DNA methylation increases LFA-1 overexpression. Acetylation of histone H4 and phosphoacetylation of H3 following proinflammatory signals result in the increased activity of NF-κB and consequent increase in the production of IL-6, the levels of which augment with age. Treatment of phytohaemagglutinin (PHA)-stimulated peripheral blood lymphocytes with a histone deacetylase (HDAC) inhibitor resulted in hypoacetylation of histone H4 with increasing age. In similarly treated mice, *in vivo *production and suppressive function of Foxp3^+^CD4^+^CD25+ regulatory T cells (T_reg_) were increased. Epigenetic mechanisms linked to ageing are believed to contribute to diminished anti-tumour immunosurveillance, either [refs. in [8]].

Both innate and adaptive immune responses are affected by age-related deficits. Deterioration of the innate immunity appears to be the prevalent mechanism associated with age-related infections, while humoral immunity retains most of its original activity throughout the life span [2,20]. It is worth remembering that innate immune responses are a first step toward the development of adaptive immune responses, and that age-related deficits in innate immune functions might therefore alter both cell-mediated and humoral adaptive immune reactions [21].

## 3. Impact of ageing on innate immunity and its restoration

Innate immunity is a key element of the immune response including several cellular components such as macrophages, NK cells, and neutrophils, which provide rapid first-line defence against pathogens. The function of these cells declines with age. Although their production increases with age, in the elderly macrophages have a reduced ability to secrete tumour necrosis factor (TNF), a key inflammatory cytokine [22]. Macrophage-derived TNF and interleukin (IL)-1 are essential for the secretion of other cytokines critical for bone marrow stromal integrity, such as IL-6, IL-11, monocyte colony stimulating factor (M-CSF), granulocyte-monocyte (GM)-CSF and receptor activators of NF-κB ligand [23]. Ageing also dampens the secretion of IL-7 by bone marrow stromal cells [24]. IL-7 is an essential survival cytokine for developing lymphocytes [25]. Furthermore, the innate immune system detects pathogens using pattern-recognition receptors such as the TLRs, which recognise specific molecular patterns present on the surface of pathogens. TLRs are expressed on a variety of cells including macrophages. Interaction between TLRs and a pathogen stimulates the secretion of a wide range of antibacterial peptides that destroy the pathogen and trigger an inflammatory response through cytokine and chemokine secretion. Studies in humans and mice have shown that TLR expression and function decline with age [1] resulting in the decreased production of pro-inflammatory cytokines and chemokines as well as in the deregulation of the adaptive immune system [1]. Modulation of the innate immune system either with TLR ligands or the products of TLR activation may enhance disease resistance, immune response and vaccine effectiveness in older persons [1]. TLR ligands may strongly enhance IL-2 production, too [26]. Similarly to what observed with other cell types, both the chemotactic and phagocytic activities of neutrophils show reduced efficacy with ageing [1,27]. Beside this reduced ability to eliminate microbes, ageing macrophages and neutrophils cannot destroy cancer cells either [2].

NK cells account for about 10-20% of peripheral blood lymphocytes. Most mature NK cells (approximately 90%) express high levels of FCγRIII (CD16) and are CD56^dim^. Proliferation of NK cells primarily occurs in the bone marrow from the same common progenitor cells as T lymphocytes. Immature NK cells undergo a serial maturation process. Finally, full functional capabilities are acquired before they are released into the circulation. Survival of mature NK cells is cytokine-dependent: IL-15 appears to prolong survival via the anti-apoptotic factor Bcl-2 [28]. NK cells also play a role in the interactions between innate and adaptive immune responses. NK cell function and dynamics may be affected by ageing. Age-related reductions in NK-cell-mediated cytotoxic activity appear to be clinically relevant as they are associated with an increased risk of infection and death in elderly subjects. In mice, basal NK cell function appears to remain intact in advanced age, whereas the inducible NK response decreases, although this effect is not observed consistently in humans [29]. This functional impairment is not related to a reduction in NK cell numbers, as this tends to be well maintained or to increase with ageing possibly due to superior preservation of telomere length. The production rate of NK cells is approximately halved in elderly people due to impaired IL-2 responsiveness. Furthermore, long-lived cells dominate within their NK cell population. Certain viral infections also affect NK cells differently from what observed in the young. For example, chronic infection with human T-lymphotropic virus-1 does not perturb NK cell dynamics in the young but decreases NK cell production in old people [8].

It is important to recall that NK cell deficiencies have been shown to predispose to Epstein-Barr virus (EBV), cytomegalovirus (CMV), varicella-zoster virus and herpes simplex virus (HSV) infections [28]. Since normal NK cell activity contributes to human health and longevity [29], the decline of these cells may offer an explanation for the increased incidence of bacterial and viral pneumonia, as well as gastrointestinal and skin infections in old age [1]. Clinicians also recognise that older individuals often have difficulties in dealing with pathogens which they had previously easily overcome, including the annual return of influenza. This infection is associated with considerable morbidity and mortality in the elderly. Those over 65 years of age account for more than 90% of the deaths from influenza and have an increased likelihood to develop complications such as pneumonia [4,30]. Epidemiological evidence reveals that older individuals are often the first to be affected by new or emerging pathogens such as West Nile virus (WNV). During an epidemic of WNV in the United States in 2002, the majority of cases occurred in patients over 50 years of age. This epidemic caused 4156 cases, of which 284 were fatal; the median age of the deceased was 78 years of age [31]. Effective vaccination may not only protect against the specific pathogen targeted, but also may result in enhanced activity of the NK cell system, which in turn may be associated with superior specific vaccine responses [32]. The ineffective protection against microbes in the muco-cutaneous barriers, including the breakdown of local immunity in the gingival and oral cavity as well as in the urinary and gastrointestinal systems that are so frequent in the elderly, may all be expressions of diminished innate immunity [5].

## 4. Changes of humoral immunity in the elderly

Haematopoietic stem cells (HSCs) give rise to all cellular components of the immune system (lymphoid and myeloid). Although the haematopoietic compartment of bone marrow decreases with age, age does not appreciably affect the number and proliferative capacity of HSCs. However, through the normal maturation process, there is a block between the pro- and pre-B cell stage. Decreased responsiveness of developing B cells to IL-7 [7], decreased V-DJ recombination of immunoglobulin (Ig) genes, decreased expression of surrogate light chain λ5, and decreased activity of the transcription factors E12 and E47 with consequent alterations in Ig heavy chain expression, all play a key role in the pro-to-pre-B cell block. In a vicious circle, aged bone marrow stromal cells have a reduced ability to support B cell expansion due to decreased IL-7 production. Peripheral B cell number also changes with age, but reports are conflicting [33,34]. Studies in mice demonstrated that, in spite of reduced input of naïve B cells to the circulation, the overall peripheral B cell number did not decline with age [33,35] due to the presence of long lived memory cell subsets [36-39]. On the contrary, there is a disagreement concerning the number of mature B lymphocytes in humans. Some showed a significant increase [39,40], others found a dramatic declination of CD27+ cells [39,41,42]. Recent careful dissection of B cell subsets have revealed that a slight increase in CD27+ memory cells plus a significant increase of anergic, exhausted memory cells with CD27 down-regulation (CD27-) filled the B immunologic space in the elderly [34,43]. Novel studies on the offsprings of centenarians have revealed that these people in their 70s and 80s had a survival advantage when compared with age-matched children of parents with average life span. In both groups a decreased B cell count was observed, however, in the centenarian offsprings, naïve B cells producing IgD (IgD+CD27-) were more abundant whereas exhausted memory cells (IgD-CD27-) did not show the increase previously had been demonstrated in healthy elderly individuals. Authors concluded that the reservoir of naïve B cells might be one of the factors that make centenarian offsprings able to keep fighting off new infections, hence prolonging their life. So, the loss of naïve B cells represents a hallmark of immunosenescence [43,44]. The quality of the humoral immune response declines with age. This change is characterised by lower antibody responses and decreased production of high-affinity antibodies. B cell proliferation declines in aged mice due to declining B cell activation and defective surface Ig/B cell receptor affinity and signalling [45]. Aged CD4^+ ^T helper cells provide an inadequate assistance in germinal centres and promote low-affinity antibody production [46] due to decreased IL-2 and IL-4 release [47]. B cell progenitors undergo maturation and differentiation in secondary lymphoid tissues, such as the spleen and lymph nodes. These organs provide a highly organised structure for T and B cells to interact with one another and with antigen presenting cells (APCs), namely dendritic cells (DCs) and macrophages. Age-associated reduction of the cortex lymphocyte cellularity and germinal centres and a parallel increase in adipose tissue indicate a decreased ability to provide the proper environment for immune reactions to take place. Ineffective cooperation of the lymphocytes within the spleen and lymph nodes and shrinkage of the germinal centres are known to occur with age. Increased frequency of B cells and memory CD4^+ ^T cells and increased expression of the senescence marker p16^INK4a ^on B cells and CD8+ T cells, accompanied by a decrease in the number of γ/δ T cells, naive CD4^+ ^T cells, CD8^+ ^T cells, and IgM producing B cells were observed in the lymph nodes of aged people [48]. The homing of immature B cells to the secondary lymphoid organs has been shown to decrease across the lifespan [49], thus reducing the chance that an antigen will be recognised by its antigen-specific B cells and possibly reducing the pool of naive B cells. This results in the loss of naive B cells and an increase in memory cells with age [49,50], dampening the ability to respond to novel antigens as the subject ages. Memory cells produced early in life remain normal [51]. In fact, B cell memory may be maintained for a very long time: e.g. individuals who survived the Spanish flu still had specific antibodies 90 years later and possessed circulating B cells that secreted binding antibodies for the haemagglutinin of the H1N1 influenza virus that caused the pandemic [52].

Ageing is associated with a shift from the Th1 to the Th2 cytokine profile in response to immune stimulation. The overproduction of Th2 cytokines could augment B cell mediated autoimmune disorders by enhancing the production of autoreactive antibodies. The percentage of naïve follicular B cell declines, whereas subsets of antigen-experienced mature B cells with longer life span increase including poly/self-reactive subtypes. These cells may be reactivated due to age-associated reduction in immune tolerance or loss of tissue integrity leading to the exposure of neo-self antigens that result in aberrant autoimmune response [8].

## 5. Age-related decline of cell-mediated immunity

The bone marrow supports the generation of T cell progenitors. Studies in aged mice have shown a reduction in the number and proliferation of early T lineage progenitors defined by surface expression of Lin^-^, CD44^+^, c-Kit^hi^, and IL-7Rα^neg/lo ^[53]. Thymocyte progenitor cells enter the thymus and through several steps differentiate into single CD4^+ ^or CD8^+ ^naive T cells, which are exported to the periphery [21]. The T cell education process is regulated by cytokines and hormones, as well as by epithelial cells and DCs, macrophages and fibroblasts that make up the thymic stroma [54]. Production and maintenance of the diverse peripheral T cell repertoire are critical to the normal function of the immune system [25]. In the elderly, there is a decrease in the diversity and functional integrity of both the CD4^+ ^and CD8^+ ^T-cell subsets, which contributes to a decreased ability to respond adequately to reinfection [55]. Age-associated changes in cell-mediated immunity strongly depend on thymic functions [1]. As an individual ages, the thymus undergoes a progressive involution, and the output of new cells falls significantly. Thymic functions gradually start decreasing from year one of life [56], but the process becomes significant from around year 40 onwards [57]. Increased levels of sex hormones might contribute to this process [58]. Both the thymic epithelial space, in which thymopoiesis occurs, and the non-epithelial, non-thymopoietic perivascular space show morphological and functional alterations. The expansion of the perivascular space (adipocytes, peripheral lymphocytes, stroma) with age results in a shift in the ratio of true thymic epithelial space to perivascular space. On the contrary, the thymic epithelial space shrinks to less than 10% of the total thymus tissue by 70 years of age. When extrapolated, data suggest that the thymus would cease to produce new T cells at approximately 105 years of age [59]. This fact might strongly contribute to limit maximum human lifespan. In addition to age-related thymic atrophy, chemotherapy, irradiation prior to transplants, septic shock and acute stress in general also lead to thymic atrophy. Deficiencies of leptin or leptin receptor in mice elicit chronic thymic atrophy [60] suggesting a key regulatory role for leptin in thymopoiesis. Leptin also protects against bacterial endotoxin-induced thymic atrophy [61]. Thymic atrophy might also result from ageing of the T cell progenitor population. Similar to what occurs in bone marrow and peripheral sites, cytokines within the thymus are crucial for thymopoiesis. Thymic epithelial cells produce a number of colony-stimulating factors and haemopoietic cytokines such as IL-1, IL-3, IL-6, IL-7, transforming growth factor (TGF)-β, oncostatin M (OSM) and leukaemia inhibitory factor (LIF). Thymic atrophy and decreased thymopoiesis are active processes mediated by the upregulation of thymosuppressive cytokines, especially IL-6, LIF and OSM in aged human and mouse thymus tissue [25,54], while IL-7 production by stromal cells significantly decreases [62]. IL-7 is necessary for thymopoiesis, promoting cell survival by maintaining the anti-apoptotic protein Bcl-2 and inducing V-DJ recombination [63]. The above changes result in the decreased thymic output of naive T cells (CD45RA^+^, CD28^+ ^and CD45RA^+^,CD28^+^,CD26L) and in the decreases concentration of these cells in peripheral blood and lymph nodes observed during ageing [25,48]; consequently, there is a shift in the ratio of naive to memory T cells in the periphery to maintain peripheral T cell homeostasis. Naive T cells from aged mice exhibit reduced activation, differentiation and cytokine production following antigen presentation [51]. Relative to memory cells generated from young naive cells, Th1 memory cells derived from aged naive cells produced much less IL-2; furthermore, Th2 memory cells derived from aged naive cells produced much less IL-4 and IL-5 [25]. Aged CD4^+ ^T cells have decreased CD40L [64], a critical co-stimulatory ligand for T-B cell interactions, due to IL-2 deficiency. Furthermore, impaired T-B cell interactions significantly contribute to the impairment of humoral responses in the aged [51]. Memory T cell activation has also been found to be attenuated, showing reduced signalling capacity and proliferation. Memory T cell function in aged mice seems to be dependent upon when the response was initiated, since memory cells produced early in life are normal [51]. Other data show that proliferative responsiveness, interferon (IFN)-γ secretion and antiviral (anti-influenza and anti E55^+ ^retroviral) capacity of cytotoxic CD8^+ ^T-cells are severely compromised as a result of ageing [65].

In the course of T-cell dependent immune responses, naive T cells must be activated by appropriate contact with APCs, especially DCs, and undergo extensive clonal expansion. Although antigen presentation by DCs generally seems to be only subtly different in the elderly in many respects, there are quantitative differences, with less numerous peripheral blood and follicular DCs [27]. Chemotaxis and phagocytosis may be impaired in DCs from the elderly. DCs from young and elderly people are reported to stimulate naive CD8^+ ^T cells equally well, but those from the elderly may fail to stimulate naive CD4^+ ^T-cells properly, perhaps due to altered signal transduction pathways [59]. These processes depend on either the number or the structure of cell surface receptors [7]. The number of TCR molecules per T cell does not change with age, however there are clear alterations in the number and balance of receptors that mediate positive or negative co-stimulatory signals [7]. There is no evidence for alterations in the actual structure of TCRs or co-stimulatory receptors with age, but it is likely that the assembly of these molecules into functional units is compromised leading to altered signal transduction. One factor that influences TCR assembly is cell membrane fluidity. The increased levels of cholesterol which occur commonly in the elderly might well contribute to age-associated deficits in T cell signalling. Elderly people possess reduced levels of tyrosine kinase Lck which is essential for T cell stimulation [66]. During maturation, thymocytes rearrange their TCR genes [25]. To mount an adequate immune response, a broad TCR repertoire must be maintained by ensuring the continuing presence of a diverse population of T cell clones, but this repertoire decreases with age. There is a corresponding reduction in the diversity of the naive TCR repertoire, which explains the decreased ability of the elderly to resist infections to which they were not previously exposed, or to respond to new antigens [7]. At least for CD4^+ ^T-cells, this may occur quite suddenly with TCR diversity well maintained up to age 60-65 years, despite marked decreases in thymic output. However, repertoire diversity in the 75-80 year old is severely reduced [55], probably contributing to the poor responses to infection and vaccination in this age group. In addition, in the elderly about 10% of T cells express the senescence marker CD57, which is instead rather infrequent in the young [1]. On the contrary, approximately 40% of naive T cells of old people do not express the T cell homing receptors CD62L and CCR7, probably implying that these cells cannot migrate properly to peripheral lymphoid tissues to encounter APCs [1].

In recent years, an important role of CD4^+^,CD25^+^, Fox3^+ ^regulatory T cells (T_reg_) in the maintenance of immune homeostasis has been described [21,67]. Characterization of these cells from young and elderly donors revealed that those in poor health conditions of either age group had significantly more T_reg _cells than their healthy counterparts. In healthy adults (aged 20-60 years) an average 0.6-8.7% of CD4^+ ^T cells are regulatory ones. Aged individuals (> 65 years) have an increase in peripheral blood T_reg _cells, but the lack of IL-7 receptor (CD127) expression on the surface of these cells results in their functional damage [68]. T_reg _lymphocytes downregulate the immune response after elimination of an antigen [6], control the host immune response to prevent damages to host tissues [69], and protect the host from self-reactive lymphocytes by deleting autoreactive immunocompetent cells. It has been suggested that a decrease in T_reg _cell numbers or function could result in autoimmune diseases or rejection of a transplant, while an excess of T_reg _lymphocytes might contribute to poor responses to infectious diseases, vaccines and cancer [69]. A major obstacle to therapeutic vaccination for chronic infections, such as human immunodeficiency virus (HIV), hepatitis C virus (HCV) and *M. tuberculosis *is to overcome the immunosuppressive effects of pathogen-specific T_reg _cells [21]. Removal of these cells using an anti-CD25 depleting antibody prior to immunization with a DNA or peptide vaccine against HSV type 1 enhanced CD8^+ ^T cell response to the virus. Depletion of CD25^+ ^cells also enhanced IFN-γ production and the level of protection induced by a malaria vaccine in mice [refs. in [21]].

## 6. Improving antigen presentation, activation of co-receptors

Reduced DC activity might represent another hurdle to be overcome in developing successful vaccination strategies for the elderly. Many active immunotherapy protocols for cancer patients rely largely on antigen presenting DCs to be recruited to the site of vaccination and take up vaccine antigen, and the same is likely to be true when immunotherapy is used to combat an infectious agent [7]. Aged CD4^+ ^T lymphocytes have decreased CD40L, a critical co-stimulatory ligand for T-B cell interactions. IL-2 enhances CD40L expression [70]. In aged individuals, the expression of CD134, a TNF receptor family member, on T cells is reduced. Co-stimulation of CD134 increases T lymphocyte function, survival, expansion and development into memory cells [71]. The increased proportion of CD8^+ ^T lymphocytes lacking expression of the co-stimulatory receptor CD28 leads to autoimmune diseases such as rheumatoid arthritis, diabetes mellitus, and multiple sclerosis, some types of cancer such as melanoma, and chronic infection with viruses such as HIV, hepatitis B virus (HBV) and CMV [refs. in [7]], decreased vaccine responsiveness and early mortality [72]. CD28 loss is the most consistent immunological marker of ageing. In pathological states, CD28 negative T cells represent prematurely senescent cells resulting from permanent immune activation [9]. These cells are potent producers of proinflammatory cytokines, acquire cytolitic capability, and have shorter telomeres than their normal counterparts [8]. The loss of CD28 expression on T cells correlates with an increased expression of CD95 and, consequently, with an accumulation of CD95^+ ^T cells. The latter show a reduced susceptibility to apoptosis, probably due to decreased production of CD95 ligand after activation and upregulation of several anti-apoptosis Bcl-2 family members [9]. T cells that have undergone clonal exhaustion after chronic viral infection also express the B7-family receptor named programmed cell death-1 (PD-1), that inhibits co-stimulatory signals. It has been suggested that blocking PD-1-mediated signalling could lead to improved T cell functions [73]. Collectively, these data indicate that stimulation via co-receptors/co-stimulatory molecules might overcome some of the intrinsic defects of immune responses in the aged [25].

## 7. Restoration of thymus functions

Reversion or block of age-related atrophy of the thymus might be one of the promising therapeutic measures to reconstitute immune functions in the elderly. It was reported that in mice transplantation of aged thymuses into juvenile recipients led to reconstruction of the structure and function of the thymus [74]. Moreover, transplantation of cultured thymic fragments to patients with DiGeorge syndrome who lack a functional thymus has been carried out successfully [74] and may also be a conceivable approach to restore naive T lymphocyte numbers in the elderly. Given the paucity of naive T cells in the elderly, enhancement of thymic output is likely to be a key requirement for maintaining and restoring their effective immunity. Neonatal thymuses grafted under the kidney capsule of mice exported considerable numbers of T cells [7]. The biological pathways that control thymic cytokine microenvironment and thymic epithelium represent candidate targets for therapies to modify age-induced thymic involution. Enhancing thymic export of T lymphocytes will theoretically increase the ability to mount successful immune responses to any antigen. Inclusion in a vaccine formulation of agents to enhance thymopoiesis may prove very useful in promoting a robust, broad T cell response to any antigen (viral, bacterial, parasite, tumour) for which a successful vaccine is needed. Reversing the decline in the immune response could also be achieved by removing senescent immune cells, therefore eliminating any potential detrimental effect deriving from these cells and permitting their replacement with naive lymphocytes through the thymic output. As seen above, there are several potential approaches to reversing thymic atrophy and increasing the number of recent thymic emigrants but very few for removing senescent cells [75]. If the elderly must rely on their memory T cells for pathogen control in later life, it becomes crucial to know whether these cells are retained and function normally [76]. In mice, depletion of dysfunctional naive T cells can readily result in their replacement by functional recent thymic emigrants [77].

## 8. Stimulation of the impaired immune system by cytokines and hormones

Artificial replacement of cytokines, chemokines, and some of the hormones having a basic role in maintaining healthy the immune system or their induction by drugs might prevent or at least partially reconstitute immune impairment in the elderly. IL-7 seems to be the most important of such factors to protect and stimulate B and T cells. The decline in IL-7 expression levels that occurs in the elderly makes it a target for therapeutic interventions to rejuvenate thymopoiesis. It has been shown that IL-7 can reverse thymic atrophy in old animals, ensuring increased thymic output to replenish the peripheral T lymphocyte pool and improving immune responses [78]. Human IL-7 administration to young mice (aged 6-8 weeks) minimally increased thymopoiesis and peripheral T cell expansion [79], but the direct subcutaneous injection of IL-7 does not result in sufficiently high concentrations of the molecule in the thymus. To overcome this problem the ideal would be targeting IL-7 to the thymus by creation of a fusion protein. The molecule CCL25 is produced in the thymus and binds to the chemokine receptor CCR9, for which it is the only known ligand. A fusion protein between the extracellular portion of CCR9 and IL-7, when used as a therapeutic agent in old animals, resulted in its accumulation in the thymus, the reversal of age-associated thymic atrophy, a significant increase in the production of new T lymphocytes, and a significant improvement in antiviral responses [80]. Mice treated with the fusion protein had a lower viral load in their lungs compared with sham-treated counterparts following influenza virus infection. Based on studies in mice and monkeys, local delivery of this cytokine to the thymus by implantation of genetically engineered stromal cells secreting IL-7 may be a way of avoiding undesired systematic effects [81]. Clearly, IL-7 has an important role in thymic functioning, and the above findings indicate that modulation of IL-7 is feasible and may offer an approach to increasing the response to vaccination in the elderly.

By studying other biological response modifiers, it has been shown that intrathymic injection of an IL-10 expressing adenovirus can prevent thymocyte apoptosis and the thymic atrophy induced by sepsis in mice [82]. Leptin protects against bacterial endotoxin-induced thymic atrophy [61]. Keratinocyte growth factor (KGF) and thymic stromal lymphopoietin (TSLP), which is known to stimulate thymus stromal cells, can promote thymopoiesis in mice following chemotherapy-induced thymic atrophy [83]. The growth hormone has also been shown to have a stimulatory effect on thymopoiesis in old mice and ablative/bone marrow transplant mice [84]. Together, these studies suggest that coupling thymostimulatory regimens such as IL-10, leptin, KGF and TSLP with inhibition of thymosuppressive cytokines [see refs. in [7]] may be efficacious in the reconstruction of an aged immune system [25]. Thymic epithelial cells, which are required to support thymocyte maturation, undergo apoptotic death in the aged thymus, through a pathway involving interactions between Fas and Fas ligands. Because age-associated thymic involution is reported not to occur in aged Fas-deficient mice [85], blocking this pathway locally in the thymus might also contribute to retaining thymic functionality. The same may apply to tumour growth factor (TGF)-β2, as greater cellularity and higher levels of naive T lymphocytes are seen in old TGF-β2-deficient mice compared to old wild-type mice [86]. IL-2 and IL-4 might restore the proliferative capacity of aged B cells [47]. Naive T cells from old animals do seem to be impaired, as CD4+ T lymphocytes show decreased helper activity and IL-2 production, but both these activities can nevertheless be partially restored by exposure to a mixture of pro-inflammatory cytokines (IL-1, IL-6, TNF) [87]. Thus, judicious local use of these cytokines as adjuvants might be beneficial. However, in elderly humans there may be vanishingly few naive cells remaining that could be targeted in this way. On the other hand, some workers recommend the opposite, because the use of anti-inflammatory agents to decrease the levels of IL-1, IL-6 and TNF may also assist in rebalancing immunity; e.g. statins are already being used extensively in the elderly to treat autoimmune responses [88]. However, the efficacy of any approach to influence inflammation is very much open to question, as inflammation also has protective effects against pathogens.

Concerning the effect of hormones on the dysfunctional aged immune system, it has been demonstrated that significant boosts in cellularity and decreased adiposity of bone marrow occurred in aged mice treated with recombinant growth hormone. In addition to its ability to stimulate aged bone marrow stroma, this hormone can also have a positive effect on early T cell lineage progenitors and stimulate thymopoiesis in aged mice, bone marrow transplant mice, and bone marrow colonised foetal thymus organ cultures [84]. Sex steroid ablation in men undergoing therapy for prostate cancer is reported to result in increased numbers of circulating naive T lymphocytes, but this approach is obviously not generally applicable to the majority of elderly people [7]. Similarly, it has been known since ancient times that eunuchs live longer than normal males, perhaps due to preserved immune functions.

## 9. Persistent microbial infections may contribute to immune exhaustion

Several environmental factors may drive T cell senescence *in vivo*, among which are infections with immunosuppressive viruses that establish latency such as CMV, EBV, HBV and HCV. These viruses can chronically stimulate T cells and may be responsible for the presence of oligoclonally expanded virus-specific CD8^+ ^T cells, the majority of which dysfunctional or anergic, in the elderly [5,7,9,89]. CMV infection may have an especially significant impact on immune parameters in later life. There is some epidemiological evidence for excess mortality in CMV seropositive populations, which is further increased in those co-infected with hepatitis A and B as well. CMV seropositive individuals also have higher levels of CRP, indicating that they are more likely to suffer with chronic inflammation. However, it is obvious that immunosenescence is not caused by CMV, because not all elderly people are CMV positive [33]. A recent study has also shown a progressive accumulation of HSV specific T cells with a central memory phenotype and exhaustion in old mice. However, continuous administration of antiviral drugs did not alter the course of this accumulation of T cells. These mice have a shorter remaining survival time than people of the same age with fewer of these cells. These finding suggests that eventual loss of control of herpesvirus infections after a lifetime of immunosurveillance may indeed be a cause of mortality in the elderly population [7,90]. It is noteworthy that analogous phenomena of clonal exhaustion and senescence may also occur in HIV infection [91]. In some respects, AIDS may also be regarded as an extremely accelerated, premature ageing. Other human pathogens may play a similar role. Human herpesvírus (HHV) 6, variants A and B, infect CD4^+ ^T lymphocytes and macrophages and establish lifelong latency and persistence in these cells. HHV-6A transactivates HIV enhancing AIDS progression and oncogenic human papillomavirus types facilitating cervical cancer development. HHV-6A, along with CMV, is frequently reactivated in immunocompromised patients, especially transplant recipients aggravating their CMV disease. HHV-6 variants are believed to be cofactors in chronic disorders such as multiple sclerosis and lymphomas frequently seen in elder people. HHV-7 infects CD4+ T lymphocytes alone, establishes life-long persistence and reactivates HHV-6. The products of early genes of HHVs and consequently, the altered cytokine release by infected cells play roles in the transactivating effect on other viruses [92]. It is conceivable that the persistent infections by these and other viruses contribute to the continuous antigenic stress and exhaustion of the immune system. In this regard, it is worth recalling that molecular techniques have recently greatly expanded the list of viruses that produce chronic productive infections in humans. A most interesting example is provided by the vast array of small unenveloped single-stranded DNA viruses that are currently classified within the family *Anelloviridae*. An amazing epidemiological feature of these newly recognized viruses is that they produce an apparently life-long high-titre, often mixed plasma viraemia in essentially all people regardless of age, health status and other variables. The prototypes of these viruses, torquetenoviruses, have been shown to replicate extensively, if not solely in haematopoietic cells, and it is believed that the other members of the family share the same tropism. Although formal proof has yet to be obtained similar to all the other viruses that lack an external cell-derived envelope, the anelloviruses are likely to be cytocidal for the cells in which they replicate. In any case, their florid replication in the haematopoietic cell compartment throughout life may represent a remarkable stress for the progenitor and precursor cells from which all the immune cells originate. In turn, even though the formidable regenerative potential of haematopoietic cells may prevent the clinical emergence of cell damage for long periods of time, in the long run this continued replication is likely to contribute significantly to the decay of immune responsiveness that becomes appreciable late in life [93]. Long-term exposure to other persistent stimulating agents, including parasite antigens especially important in developing countries, may yield similar effects [7].

Strategies to reduce the chronic infectious antigenic load would seem to offer a reasonable approach to restoring appropriate immune functions and might also benefit naive T cell production. Targeting these viruses and any other infectious agents that have established a persistent infection may be of clinical benefit both directly by reducing the pathological consequences of the infections and in terms of improving responses to vaccinations. Unfortunately, the continuous administration of antiviral drugs did not alter the course of progressive accumulation of virus-specific exhausted memory T cells in old mice [7]. As CMV persistency and influenza virus infection may exert especially detrimental effects on the immune system in the elderly, studies have focused on attempts to eliminate their effects. The putatively dysfunctional CMV-specific CD8^+^CD28^+ ^T lymphocytes that accumulate in the elderly and that seem to be anergic and apoptosis-resistant may be restored to functional competence directly ex vivo by culturing them with IL-2. In this respect, they behave like anergic T cells that can be found in many experimental situations of chronic antigen exposure; notably, in at least some such situations several approaches, including the blocking of inhibitory receptors such as PD-1, can restore T lymphocyte functions [94]. One option, again, could therefore be to treat the elderly with recombinant IL-2: an early study reported that elderly people given well tolerated low doses of IL-2 just before receiving influenza virus vaccination produced higher antibody titres and were better protected then controls vaccinated without IL-2 pre-treatment [95]. A recent study using a novel IL-2 supplemented liposomal influenza vaccine in a group of elderly people also found superior responses with the use of IL-2 [96].

## 10. Problems and new possibilities with vaccination in the elderly

One of the greatest practical health-care challenges in the elderly is to ensure that vaccinations are optimally effective. Increasing the efficacy of vaccination would have an enormous impact on health and well-being [7]. Development of vaccination strategies that are effective in all age groups is an important area of research, particularly to protect against new or re-emerging fatal infections and possible infectious agents that might be weaponised by bioterrorists [97]. Investigations on the adequateness of responses to vaccination require longitudinal studies following the same individuals over time and correlating test parameters with clinical outcome (including responses to the vaccine, pathology and disease-specific lethality). Better vaccine efficacy in the elderly may require a two-pronged attack on the problem, consisting of an improvement in the immune responsiveness and an alteration to vaccine formulations. Vaccination can only be effective if cells that are capable of responding are still present in the repertoire [7]. Unfortunately, immunosenescence also compromises the response to vaccination. Since the elderly comprise the largest target population for influenza vaccination, the majority of studies evaluating the efficacy and benefits of vaccination have been conducted among people in this age group [98]. Influenza is the fifth leading cause of death in the developed world among people aged 50 and older, and this group is the major target of vaccination campaigns [4,99]. This vaccination strategy is controversial, as it appears to have only a moderate impact on influenza-specific immune responses and infection rates in this group [100]. While influenza vaccination has 70-90% efficacy in healthy adults in Western countries, the success rate falls to 17-53% in the elderly [101] when determined as specific immune responses. Further data show that in people aged 65 years and over, effectiveness measured by the prevention of influenza-like illness is 23%, while in healthy children (aged > 2 years) the effectiveness is higher, at 38% [20]. The effect of influenza vaccination on the risk of pneumonia in elderly people during influenza seasons might also be less than previously estimated [4]. The reasons for these unsatisfactory outcomes of vaccination have been clearly established: both the humoral and cell-mediated influenza-specific responses are lower than in young adults [98]. Peripheral blood mononuclear cells from elderly adults (> 65 years) stimulated with influenza A virus *ex vivo *exhibited a decrease in IFN-γ^+ ^T cells and in the secretion of IFN-γ by individual cells when compared to young adults (aged 20-50 years). Expansion of CD8^+ ^T cells was also reduced in the aged, who also produced fewer antibodies in response to influenza virus [102]. In mouse models, influenza virus is cleared from the lungs more slowly in old than in young animals, apparently due to decreased cytotoxic T cell production. Cell transfer experiments demonstrated that both the old cells and the old microenvironment of cells contribute to the problem; indeed, even young functional T cells did not respond properly when transferred to an old individual, and neither did old cells when transferred to a young subject [103]. Patient-individualised strategies will have to take the immunological age of the person closely into account. For this, it will be necessary to differentiate between chronological and functional age of immunity, an assessment that could be based on a constellation of immune markers [7]. In the case of new influenza vaccines targeted for use in the elderly, improvement of the formulations are primarily sought at the level of T cell immunity [98]. During the course of an influenza virus infection, bacterial superinfections often represent the ultimate determinant of morbidity and mortality. Post-mortem examination of fatal confirmed pandemic H1N1 2009 influenza demonstrated evidence of coexistent bacterial infection in nearly one third of cases. An impairment of the TLRs and/or APCs can be pivotal in the genesis of these bacterial complications [104].

The second prong on the assault on the problem would be to develop vaccines especially designed for the elderly. Several strategies have been explored, including the use of high dose of the immunogens [105], DNA vaccines with immunostimulatory patch [106], recombinant virus-like particles (e.g. the vaccine against human papilloma viruses [6]), virosomal vaccines [107], and adjuvant vaccines [108]. Virosomes represent a novel vaccine presentation form that closely mimic a native virus. They are virus-like particles consisting of reconstituted viral envelopes or capsids lacking the viral genetic material and bind and fuse to different target cells including immune cells. Virosomes can deliver encapsulated protein antigens or even large transfecting plasmid DNA to the cytosol. Protein antigens might thus bypass the potential problem of MHC restriction and induce strong CTL responses. Foreign antigens that may be coupled to the surface of virosomes can be recognised by membrane-associated Ig receptor molecules on B lymphocytes. Influenza-derived virosomes stimulated co-stimulatory signaling, for example B7.1 (CD80), B7.2 (CD86), CD40, MHC class I and class II expression on DCs. Intact virions of inactivated hepatitis A virus (HAV) were non-covalently coupled to the surface of influenza virosomes, and this combined vaccine induced seroconversion in 100% with high and long-lasting HAV-specific antibody titres [98]. Virosomes containing amphiphilic adjuvants incorporated into their membrane offer the additional advantage of combining APCs activation with presentation of the antigen by the same cells. A combined influenza virosome vaccine was recognised by TLR-2, which is present on B lymphocytes and DCs. In mice, a single injection resulted in a 150-fold increased IgG response compared to virosomes lacking the adjuvant [98]. Adjuvanted influenza vaccines, such as those containing oil-in-water emulsion (e.g. squalene, Tween 80 and sorbitan triolerate [98]), will have an important role, because they have been shown to induce stronger and more effective serologic responses in the elderly than conventional non-adjuvanted vaccines, not only against homologous but also against heterovariant strains [108]. The H5N1 pre-pandemic influenza virus strain administered with an oil-in-water adjuvant emulsion generated a more robust immune response compared to the non-adjuvanted version in a preclinical trial [104]. Adjuvants may also reduce the dose requirements for vaccine antigen, a factor of particular importance in the context of a much increased demand for vaccine in a pandemic situation [98,104]. Furthermore, a combination of improved vaccines with better adjuvants and immunostimulatory agents would be of increased benefit, as the commonly applied adjuvant alum is only marginally effective in the elderly [109]. A pandemic H1N1 2009 inactivated influenza virus strain adjuvanted with alum enhanced immunogenicity only moderately [104]. Indeed, alum mainly enhances antibody responses, whereas resistance to viruses and cancer may benefit from enhanced cell-mediated immunity. Adjuvanted vaccines strongly support the notion that superior vaccines can be designed with the aim of overcoming immunosenescence and/or improving protection in the elderly population [1]. Interestingly, TLR ligands can direct the immune response into specific directions; therefore, they also have a great potential as adjuvants. TLR agonists can block the anti-inflammatory arm of the innate immune responses and are rapidly emerging as potential adjuvants for enhancing the immunogenicity of subunit vaccines. When applied to infectious disease and tumour models, the adjuvant combination of a TLR agonist and a p38 MAPK inhibitor suppressing IL-10 and prostaglandin E_2 _production by DCs, considerably enhanced vaccine efficacy, selectively inhibiting the T_reg _promoting arm of innate immunity [6,21]. Much work is also focused on optimising delivery systems for TLR ligands to enhance their adjuvanticity [6].

The cancer vaccines that are currently under development differ from the vast majority of those against infectious diseases, because they are administered after the onset or detection of disease, thus they are correctly regarded as therapeutic rather than prophylactic vaccines. They contain tumour cell lysates or defined cancer antigens. The latter vaccines are usually a single antigen that is delivered by one of a number of delivery systems that include recombinant proteins and viral vectors, peptides, or *ex vivo *treated DCs. Another new field is the vaccine-like approach that is being explored as a means of containing Alzheimer disease. Since the plaques deposited in the brain of patients originate from a neuronal membrane-bound protein, the amyloid precursor protein, experimental vaccines consist of analogues of the β-amyloid peptide coupled to a carrier. The aim is to raise antibodies which recognise plaques, Aβ deposits and β-amyloid in brain blood vessels, but do not see the amyloid precursor protein or Aβ [6].

## 11. Concluding remarks

Frequent and severe microbial infections influence the quality of life of elderly individuals and their families, and have a deleterious financial impact onto the health-care system. Theoretically, several types of infectious diseases might be prevented by vaccinating old people. Lately it has become evident that vaccination or re-vaccination in this age group is not only a political agenda or financial matter, because age-related alterations of the immune system would render most vaccines ineffective in the majority of subjects. An improved understanding of immune dysfunction in human ageing will increase the probability of discovering means to restore appropriate function and alleviate the burden of infectious diseases later in life. Prevention of immunosenescence or at least partial reconstitution of the immune activities are required if a vaccination regimen is introduced in these people, who very often suffer of multiple important comorbidities. Experimental data and clinical observations outlined above demonstrate that both innate and adaptive immunity can be improved in the elderly. Any successful intervention might possibly boost other immune reactions due to several feed-back mechanisms. Activation of the exhausted immune system by using cytokines and judicious application of hormones appear to be promising practical approaches to combat chronic infection with immunosuppressive persistent viruses. Furthermore, new types of vaccine formulation, especially virosomal vaccines and adjuvanted vaccines, permit improved antigen presentation and cytotoxic activity against intracellular pathogens. Hopefully, at least some of the approaches outlined here will be translated to human medicine in a not too far future.

## Competing interests

The authors declare that they have no competing interests.

## Authors' contributions

Both authors drafted the manuscript and approved the text.

## References

[B1] Aspinall R Del Giudice G Effros RB Grubeck-Loebenstein B Sambhara S Challenges for vaccination in the elderly Immun Ageing 2007 4 9 18 10.1186/1742-4933-4-9 18072962 PMC2235886

[B2] Bulati M Pellicanò M Vasto S Colonna-Romano G Understanding ageing: Biomedical and bioengineering approaches, the immunologic view Immun Ageing 2008 5 9 11 10.1186/1742-4933-5-9 18768084 PMC2542987

[B3] Gavazzi G Krause KH Ageing and infection Lancet Infect Dis 2002 2 659 66 10.1016/S1473-3099(02)00437-1 12409046

[B4] Jackson ML Nelson JC Weiss NS Neuzil KM Barlow W Jackson LA Influenza vaccination and risk of community-acquired pneumonia in immunocompetent elderly people: population-based, nested case-control study Lancet 2008 372 398 405 10.1016/S0140-6736(08)61160-5 18675690

[B5] Crétel E Veen I Pierres A Bongrand P Gavazzi G Immunosénescence et infections, mythe ou réalité? Med Mal Infect 2010 doi: 10.1016/j.medmal.2009.12.008 10.1016/j.medmal.2009.12.008 20092974

[B6] Boog CJP Principles of vaccination and possible development strategies for rational design Immunol Lett 2009 122 104 7 10.1016/j.imlet.2008.11.009 19100778

[B7] Derhovanessian E Solana R Larbi A Pawelec G Immunity ageing and cancer Immun Ageing 2008 5 11 27 10.1186/1742-4933-5-11 18816370 PMC2564902

[B8] Grolleau-Julius A Ray D Yung RL The role of epigenetics in aging and autoimmunity Clinic Rev Allerg Immunol 2009 doi: 10.1007/s12016-009-8169-3 10.1007/s12016-009-8169-3 PMC2889224 19653133

[B9] Gharagozloo M Bagherpour B Tahanian M Oreizy F Amirghofran Z Sadeghi HMM Premature senescence of T lymphocytes from patients with β-thalassemia major Immunol Lett 2009 122 84 88 10.1016/j.imlet.2008.12.003 19118576

[B10] Dagarag M Evazyan T Rao N Effros RB Genetic manipulation of telomerase in HIV-specific CD8+ T cells: enhanced antiviral functions accompany the increased proliferative potential and telomere length stabilization J Immunol 2004 173 6303 11 15528369 10.4049/jimmunol.173.10.6303

[B11] Sliedrecht A den Elzen WPJ Verheij JM Westendorp RGJ Gussekloo J Incidence and predictive factors of lower respiratory tract infections among the very elderly in the general population The Leiden 85-plus study. Thorax 2008 63 817 22 10.1136/thx.2007.093013 18388206

[B12] Ritz BV Gardner EM Malnutrition and energy restriction differentially affect viral immunity J Nutr 2006 136 1141 44 16614394 10.1093/jn/136.5.1141

[B13] Marko MG Ahmed T Bunnell SC Wu D Chung H Huber BT Age-associated decline in effective immune synapse formation of CD4^+ ^T cells is reversed by vitamin E supplementation J Immunol 2007 178 1443 49 17237392 10.4049/jimmunol.178.3.1443

[B14] Franceschi C Capri M Monti D Giunta S Olivieri F Sevini F Inflammaging and anti-inflammaging: a systemic perspective on aging and longevity emerged from studies in humans Mech Ageing Dev 2007 128 92 105 10.1016/j.mad.2006.11.016 17116321

[B15] Albers R Wielen RP van der Brink EJ Hendriks HF Dorovska-Taran VN Mohede IC Effects of cis-9, trans-11 and trans-10, cis-12 conjugated linoleic acid (CLA) isomers on immune function in healthy men Eur J Clin Nutr 2003 57 595 603 10.1038/sj.ejcn.1601585 12700622

[B16] Larbi A Douziech N Dupuis G Khalil A Pelletier H Guerard KP Age-associated alterations in the recruitment of signal-transduction proteins to lipid rafts in human T lymphocytes J Leukoc Biol 2004 75 373 81 10.1189/jlb.0703319 14657209

[B17] Duffy D Rader DJ Drugs in development: targeting high-density lipoprotein metabolism and reverse cholesterol transport Curr Opin Cardiol 2005 20 301 6 10.1097/01.hco.0000168532.69342.26 15956827

[B18] Effros RB Walford RL Weindruch R Mitcheltree C Influences of dietary restriction on immunity to influenza in aged mice J Gerontol 1991 46 B142 B147 2071828 10.1093/geronj/46.4.b142

[B19] Messaoudi I Warner J Fischer M Park B Hill B Mattison J Delay of T cell senescence by caloric restriction in aged long-lived nonhuman primates Proc Natl Acad Sci USA 2006 03 19448 53 10.1073/pnas.0606661103 PMC1748246 17159149

[B20] Scrimshaw NS San Giovanni JP Synergism of nutrition, infection, and immunity: an overview Am J Clin Nutr 1997 66 464S 477S 9250134 10.1093/ajcn/66.2.464S

[B21] Mills KHG Designer adjuvants for enhancing the efficacy of infectious disease and cancer vaccines on suppression of regulatory T cells induction Immunol Lett 2009 122 108 11 10.1016/j.imlet.2008.11.007 19100777

[B22] Wang CQ Udupa KB Xiao H Lipschitz DA Effect of age on marrow macrophage number and function Aging (Milano) 1995 7 379 84 8719605 10.1007/BF03324349

[B23] Rebel VI Miller CL Eaves CJ Lansdorp PM The repopulation potential of fetal liver hematopoietic stem cells in mice exceeds that of their liver adult bone marrow counterparts Blood 1996 87 3500 507 8605370

[B24] Tsuboi I Morimoto K Hirabayashi Y Li GX Aizawa S Mori KJ Kanno J Senescent B lymphopoiesis is balanced in suppressive homeostasis: decrease in interleukin-7 and transforming growth factor-beta levels in stromal cells of senescence-accelerated mice Exp Biol Med 2004 229 494 502 10.1177/153537020422900607 15169968

[B25] Gruver AL Hudson LL Sempowski GD Immunosenescence and ageing J Pathol 2007 211 144 56 10.1002/path.2104 17200946 PMC1931833

[B26] Qin W Jiang J Chen Q Yang N Wang Y Wei X CpG ODN enhances immunization effects of hepatitis B vaccine in aged mice Cell Mol Immunol 2004 1 148 52 16212903

[B27] Solana R Pawelec G Tarazona R Aging and innate immunity Immunity 2006 24 491 94 10.1016/j.immuni.2006.05.003 16713963

[B28] Zhang Y Wallace DL de Lara CM Ghattas H Griffin GE Taylor GP In vivo kinetics of human natural killer cells: the effects of ageing and acute and chronic viral infection Immunology 2007 121 258 65 10.1111/j.1365-2567.2007.02573.x 17346281 PMC2265941

[B29] Murasko D Gardner EM Hazzard WR, Blass JP, Halter JB, Ouslander JG, Tinetti ME Immunology of aging Principles of geriatric medicine and gerontology 2003 5 New York NY: McGraw-Hill 35 51

[B30] Hannoun C Megas F Piercy J Immunogenicity and protective efficacy of influenza vaccination Virus Res 2005 103 133 138 10.1016/j.virusres.2004.02.025 15163501

[B31] O'Leary DR Martin AA Montgomery SP Kipp AM Lehman JA Biggerstaff BJ The epidemic of West Nile virus in the United States, 2002 Vector Borne Zoonotic Dis 2004 4 61 70 10.1089/153036604773083004 15018774

[B32] Mysliwska J Trzonkowski P Szmit E Brydak LB Machala M Mysliwski A Immunomodulating effect of influenza vaccination in the elderly differing in health status Exp Gerontol 2004 39 1447 58 10.1016/j.exger.2004.08.005 15501014

[B33] Caruso C Buffa S Candore G Colonna-Romano G Dunn-Walters D Kipling D Pawelec G Mechanisms of immunosenescence Immun Ageing 2009 6:10. doi: 10.1186/1742-4933-6-10 10.1186/1742-4933-6-10 PMC2723084 19624841

[B34] Colonna-Romano G Bulati M Aquino A Pellicanò M Vitello S Lio D Candore G Caruso C A double-negative (IgD^-^CD27^-^) B cell population is increased in the peripheral blood of elderly people Mech Ageing Dev 2009 130 681 690 10.1016/j.mad.2009.08.003 19698733

[B35] Weksler ME Szabó P The effect of age on the B-cell repertoire J Clin Immun 2000 20 240 249 10.1023/A:1006659401385 10939711

[B36] Aw D Silva AB Palmer DP Immunosenescence: emerging challenges for an ageing population Immunology 2007 120 435 446 10.1111/j.1365-2567.2007.02555.x 17313487 PMC2265901

[B37] Caruso C Candore G Colonna-Romano G Lio D Franceschi C Inflammation and life-span Science 2005 14 208 209 10.1126/science.307.5707.208 15655081

[B38] Gibson KL Wu YC Barnett Y Duggan O Vaughan R Kondeatis E Nilsson BO Wikby A Kipling D Dunn-Walters DK B cell diversity decreases in old age and is correlated with poor health status Ageing Cell 2009 8 18 25 10.1111/j.1474-9726.2008.00443.x PMC2667647 18986373

[B39] Kline GH Hayden TA B cell maintenance in aged mice reflects both increased B cell longevity and decreased B cell generation J Immunol 1999 162 3342 3349 10092788

[B40] Agematsu K Hokibara S Nagumo H Komiyama A CD27: a memory B-cell marker Immunol Today 2000 21 204 206 10.1016/S0167-5699(00)01605-4 10782048

[B41] Frasca D Landin AM Riley RL Blomberg BB Mechanisms for decreased function of B cells in aged mice and humans J Immunol 2008 180 2741 2746 18292491 10.4049/jimmunol.180.5.2741

[B42] Shi Y Yamazaki T Okubo Y Uehara Y Sugane K Agematsu K Regulation of aged humoral immune defense against pneumococcal bacteria by IgM memory B cell J Immunol 2005 175 3262 3267 16116217 10.4049/jimmunol.175.5.3262

[B43] Colonna-Romano G Bulati M Aquino A Pellicanò M Vitello S Lio D Candore G Caruso C A double-negative (IgD^-^CD27^-^) B cell population is increased in the peripheral blood of elderly people Mech Ageing Dev 2009 130 681 690 10.1016/j.mad.2009.08.003 19698733

[B44] Colonna-Romano G Buffa S Bulati M Candore G Lio D Pellicanò M Vasto S Caruso C B cells compartment in centenarian offspring and old people Curr Pharm Des 2010 16 604 608 10.2174/138161210790883750 20388070

[B45] Whisler RL Grants IS Age-related alterations in the activation and expression of phosphotyrosine kinases and protein kinase C (PKC) among human B cells Mech Ageing Dev 1993 71 31 46 10.1016/0047-6374(93)90033-N 8309282

[B46] Song H Price PW Cerny J Age-related changes in antibody repertoire: contribution from T cells Immunol Rev 1997 160 55 62 10.1111/j.1600-065X.1997.tb01027.x 9476665

[B47] Whisler RI Williams JW Jr Newhouse YG Human B cell proliferative responses during aging. Reduced RNA synthesis and DNA replication after signal transduction by surface immunoglobulins compared to B cell antigenic determinants CD20 and CD40 Mech Ageing Dev 1991 61 209 22 10.1016/0047-6374(91)90018-U 1726699

[B48] Lazuardi L Jenewein B Wolf AM Pfister G Tzankov A Grubeck-Loebenstein B Age-related loss of naive T cells and dysregulation of T cell/B cell interactions in human lymph nodes Immunology 2005 114 37 43 10.1111/j.1365-2567.2004.02006.x 15606793 PMC1782064

[B49] Miller RA The aging immune system: primer and prospectus Science 1996 273 70 74 10.1126/science.273.5271.70 8658199

[B50] Macallan DC Wallace DL Zhang Y Ghattas H Asquith B de Lara C Worth A B-cell kinetics in humans: rapid turnover of peripheral blood memory cells Blood 2005 105 3633 40 10.1182/blood-2004-09-3740 15644412

[B51] Haynes L Eaton SM Burns EM Randall TD Swain SL Newly generated CD4 T cells in aged animals do not exhibit age-related defects in response to antigen J Exp Med 2005 201 845 51 10.1084/jem.20041933 15781577 PMC2213095

[B52] Yu X Tsibane T McGraw PA House FS Keefer CJ Hicar MD Neutralizing antibodies derived from the B cells of 1918 influenza pandemic survivors Nature 2008 455 532 36 10.1038/nature07231 18716625 PMC2848880

[B53] Yuan R Astle CM Harrison DE genetic regulation of hematopoietic stem cell exhaustion during development and growth Exp Hematol 2005 33 243 50 10.1016/j.exphem.2004.10.014 15676219

[B54] Sempowski GD Hale LP Sundy JS Massey JM Koup RA Douek DC Leukemia inhibitory factor, oncostatin M, IL-6 and stem cell factor mRNA expression in human thymus increases with age and is associated with thymic atrophy J Immunol 2000 164 2180 87 10657672 10.4049/jimmunol.164.4.2180

[B55] Naylor K Li G Vallejo AN Lee WW Koetz K Bryl E The influence of age on T cell generation and TCR diversity J Immunol 2005 174 7446 52 15905594 10.4049/jimmunol.174.11.7446

[B56] Steinmann GG Changes in the human thymus during aging Curr Top Pathol 1986 75 43 88 3514161 10.1007/978-3-642-82480-7_2

[B57] Hakim FT Memon SA Cepeda R Jones EC Chow CK Kasten-Sportes C Age-dependent incidence, time course, and consequences of thymic renewal in adults J Clin Invest 2005 115 930 39 15776111 10.1172/JCI22492 PMC1064981

[B58] Sutherland JS Goldberg GL Hammett MV Uldrich AP Berzins SP Heng TS Activation of thymic regeneration in mice and humans following androgen blockade J Immunol 2005 175 2741 53 16081852 10.4049/jimmunol.175.4.2741

[B59] George AJ Ritter MA Thymic involution with ageing: obsolescence or good housekeeping? Immunol Today 1996 17 267 72 10.1016/0167-5699(96)80543-3 8962629

[B60] Madiehe AM Mitchell TD Harris RB Hyperleptinemia and reduced TNF-alpha secretion cause resistance of db/db mice to endotoxin Am J Physiol Regul Integr Comp Physiol 2003 284 R763 70 12571077 10.1152/ajpregu.00610.2002

[B61] Hick RW Gruver AL Ventevogel MS Haynes BF Sempowski GD Leptin selectively augments thymopoiesis in leptin deficiency and lipopolysaccharide-induced thymic atrophy J Immunol 2006 177 169 76 16785512 10.4049/jimmunol.177.1.169 PMC1993881

[B62] Andrew D Aspinall R Age-associated thymic atrophy is linked to a decline in IL-7 production Exp Gerontol 2002 37 455 63 10.1016/S0531-5565(01)00213-3 11772533

[B63] Kim K Le CK Sayers TJ Muegge K Durum SK The trophic action of IL-7 on pro-T cells: inhibition of apoptosis of pro-T1, -T2 and -T3 cells correlates with Bcl-2 and Bax levels and is independent of Fas and p53 pathways J Immunol 1998 160 5735 41 9637482

[B64] Eaton SM Burns EM Kusser K Randall TD Haynes L Age-related defects in CD4 T cell cognate helper function lead to reductions in humoral responses J Exp Med 2004 200 1613 22 10.1084/jem.20041395 15611289 PMC2211991

[B65] el Rafaei M Blank KJ Murasko DM Prolonged E55^+ ^retrovirus expression in aged mice is associated with a decline in the antivirus immune response Virology 2001 290 281 89 10.1006/viro.2001.1128 11883192

[B66] Tewari K Walent J Svaren J Zamoyska R Suresh M Differential requirement for Lck during primary and memory CD8+ T cell responses Proc Natl Acad Sci USA 2006 103 16388 93 10.1073/pnas.0602565103 17060632 PMC1637592

[B67] Wing K Suri-payer E Rudin A CD4^+^CD25^+^-regulatory T cells from mice to man Scan J Immunol 2005 62 1 15 10.1111/j.1365-3083.2005.01634.x 16091121

[B68] Liu W Putnam AL Xu-Yu Z Szot GL Lee MR Zhu S CD127 expression inversely correlates with FoxP3 and suppressive function of human CD4^+ ^T reg cells J Exp Med 2006 203 1701 11 10.1084/jem.20060772 16818678 PMC2118339

[B69] Rouse BT Sarangi PP Suvas S Regulatory T cells in virus infections Immunol Rev 2006 212 272 86 10.1111/j.0105-2896.2006.00412.x 16903920

[B70] Foy TM Aruffo A Bajorath J Buhlmann JE Noelle RJ Immune regulation by CD40 and its ligand GP39 Annu Rev Immunol 1996 14 591 617 10.1146/annurev.immunol.14.1.591 8717526

[B71] Murata S Ladle BH Kim PS Lutz ER Wolpoe ME Ivie SE OX40 co-stimulation synergizes with GM-CSF whole-cell vaccination to overcome established CD8+ T cell tolerance to an endogenous tumor antigen J Immunol 2006 176 974 83 16393983 10.4049/jimmunol.176.2.974

[B72] Effros RB Role of T lymphocyte replicative senescence in vaccine efficacy Vaccine 2007 25 599 604 10.1016/j.vaccine.2006.08.032 17014937

[B73] Riley JL June CH The road to recovery: translating PD-1 biology into clinical benefit Trends Immunol 2007 28 48 50 10.1016/j.it.2006.12.001 17188572

[B74] Markert ML Boeck A Hale LP Kloster AL McLaughlin TM Batchvarova MN Transplantation of thymus tissue in complete DiGeorge syndrome N Engl J Med 1999 341 1180 89 10.1056/NEJM199910143411603 10523153

[B75] Pawelec G Immunosenescence and vaccination Immun Ageing 2005 2 16 19 10.1186/1742-4933-2-16 16307681 PMC1315345

[B76] Herndler-Brandstetter D Schwaiger S Veel E Fehrer C Cioca DP Almanzar G CD25-expressing CD8+ T cells are potent memory cells in old age J Immunol 2005 175 1566 74 16034095 10.4049/jimmunol.175.3.1566

[B77] Clise-Dwyer K Huston GE Buck AL Duso DK Swain SL Environmental and intrinsic factors lead to antigen unresponsiveness in CD4^+ ^recent thymic emigrants from aged mice J Immunol 2007 178 1321 31 17237378 10.4049/jimmunol.178.3.1321

[B78] Andrew D Aspinall R IL-7 and not stem cell factor reverses both the increase in apoptosis and the decline in thymopoeisis seen in aged mice J Immunol 2001 166 1524 30 11160192 10.4049/jimmunol.166.3.1524

[B79] Sempowski GD Gooding ME Liao HX Le PT Haynes BF T cell receptor excision circle assessment of thymopoiesis in aging mice Mol Immunol 2002 38 841 48 10.1016/S0161-5890(01)00122-5 11922942

[B80] Henson SM Snelgrove R Hussell T Wells DJ Aspinall R An IL-7 fusion protein that shows increased thymopoietic ability J Immunol 2005 175 4112 18 16148161 10.4049/jimmunol.175.6.4112

[B81] Phillips JA Brondstetter TI English CA Lee HE Virts EL Thoman ML IL-7 gene therapy in aging restores early thymopoiesis without reversing involution J Immunol 2004 173 4867 74 15470027 10.4049/jimmunol.173.8.4867

[B82] Oberholzer C Oberholzer A Bahjat FR Minter RM Tannahill CL Abouhamaze A Targeted adenovirus-induced expression of IL-10 decreases thymic apoptosis and improves survival in murine sepsis Proc Natl Acad Sci USA 2001 98 11503 08 10.1073/pnas.181338198 11553765 PMC58759

[B83] Min D Panoskaltsis-Mortari A Kuro O Hollander GA Blazar BR Weinberg KI Sustained thymopoiesis and improvement in functional immunity induced by exogenous KGF administration in murine models of aging Blood 2007 109 2529 37 10.1182/blood-2006-08-043794 17138819 PMC1852207

[B84] Chen BJ Cui X Sempowski GD Chao NJ Growth hormone accelerates immune recovery following allogeneic T cell-depleted bone marrow transplantation in mice Exp Hematol 2003 31 953 98 10.1016/S0301-472X(03)00196-6 14550811

[B85] Yajima N Sakamaki K Yonehara S Age-related thymic involution is mediated by Fas on thymic epithelial cells Int Immunol 2004 16 1027 35 10.1093/intimm/dxh104 15192051

[B86] Kumar R Langer JC Snoeck H Transforming growth factor-beta2 is involved in quantitative genetic variation in thymic involution Blood 2006 107 1974 79 10.1182/blood-2005-04-1495 16282338 PMC1895709

[B87] Haynes L Eaton SM Burns EM Rincon M Swain SL Inflammatory cytokines overcome age-related defects in CD4 T cell responses in vivo J Immunol 2004 172 5194 99 15100256 10.4049/jimmunol.172.9.5194 PMC3752606

[B88] Foody JM Rathore SS Galusha D Masoudi FA Havranek EP Radford MJ Hydroxymethylglutaryl-CoA reductase inhibitors in older persons with acute myocardial infarction: evidence for an age-statin interaction J Am Geriatr Soc 2006 54 421 30 10.1111/j.1532-5415.2005.00635.x 16551308 PMC2797316

[B89] Pawelec G Akbar A Caruso C Solana R Grubeck-Loebenstein B Wikby A Human immunosenescence: is it infectious? Immunol Rev 2005 205 257 68 10.1111/j.0105-2896.2005.00271.x 15882359

[B90] Lang A Brien JD Messaoudi I Nikolich-Zugich J Age-related dysregulation of CD8+ T cell memory specific for a persistent virus is independent of viral replication J Immunol 2008 180 4848 4857 18354208 10.4049/jimmunol.180.7.4848 PMC4161215

[B91] Appay V Almeida JR Sauce D Autran B Papagno L Accelerated immune senescence and HIV-1 infection Exp Gerontol 2007 42 432 37 10.1016/j.exger.2006.12.003 17307327

[B92] Caselli E Di Luca D Molecular biology and clinical associations of Roseoloviruses human herpesvírus 6 and human herpesvírus 7 New Microbiologica 2007 30 173 187 17802896

[B93] Maggi F Bendinelli M Immunobiology of the torque teno viruses and other anelloviruses. Curr. Topics Microbiol Immunol 331 2009 65 90 10.1007/978-3-540-70972-5_5 19230558

[B94] Blank C Mackensen A Contribution of the PD-L1/PD-1 pathway to T-cell exhaustion: an update on implications for chronic infections and tumor evasion Cancer Immunol Immunother 2007 56 739 45 10.1007/s00262-006-0272-1 17195077 PMC11030209

[B95] Provinciali M Di Stefano G Colombo M Della Croce F Gandolfi MC Daghetta L Adjuvant effect of low-dose interleukin-2 on antibody response to influenza virus vaccination in healthy elderly subjects Mech Ageing Dev 1994 77 75 82 10.1016/0047-6374(94)90016-7 7745993

[B96] Ben-Yehuda A Joseph A Barenholz Y Zeira E Even-Chen S Louria-Hayon I Immunogenicity and safety of a novel IL-2-supplemented liposomal influenza vaccine (INFLUSOMEVAC) in nursing-home residents Vaccine 2003 21 3169 78 10.1016/S0264-410X(03)00251-2 12804845

[B97] Swain GR Ransom J Universal influenza vaccination recommendations: local health department perspectives J Publ Health Manag Pract 2006 12 317 20 10.1097/00124784-200607000-00003 16775527

[B98] Wilschut J Influenza vaccines: The virosome concept Immunol Lett 2009 122 118 21 10.1016/j.imlet.2008.11.006 19100779

[B99] Bridges CB Winquist AG Fukuda K Cox NJ Singleton JA Strikas RA Prevention and control of influenza: recommendations of the Advisory Committee on Immunization Practices (ACIP) Morb Mortal Wkly Rep Recomm Rep 2000 49 1 38 15580733

[B100] de Jong JC Beyer WE Palache AM Rimmelzwaan GF Osterhaus AD Mismatch between the 1997/1998 influenza vaccine and the major epidemic A(H3N2) virus strain as the cause of an inadequate vaccine-induced antibody response to this strain in the elderly J Med Virol 2000 61 94 99 10.1002/(SICI)1096-9071(200005)61:1<94::AID-JMV15>3.0.CO;2-C 10745239

[B101] Goodwin K Viboud C Simonsen L Antibody response to influenza vaccination in the elderly. A quantitative review Vaccine 2006 24 1159 69 10.1016/j.vaccine.2005.08.105 16213065

[B102] Deng Y Jing Y Campbell AE Gravenstein S Age-related impaired type 1 T cell responses to influenza: reduced activation ex vivo, decreased expansion in CTL culture in vitro, and blunted response to influenza vaccination in vivo in the elderly J Immunol 2004 172 3437 46 15004143 10.4049/jimmunol.172.6.3437

[B103] Katz JM Plowden J Renshaw-Hoelscher M Lu X Tumpey TM Sambhara S Immunity to influenza: the challenges of protecting an aging population Immunol Res 2004 29 113 24 10.1385/IR:29:1-3:113 15181275

[B104] Joshi SR Shaw A Quagliarello VJ Pandemic influenza H1N1 2009, innate immunity, and the impact of immunosenescence on influenza vaccine Yale J Biol Med 2009 82 143 151 20027279 PMC2794489

[B105] Couch RB Winokur P Brady R Belshe R Chen WH Cate TR Safety and immunogenicity of a high dosage trivalent influenza vaccine among elderly subjects Vaccine 2007 25 7656 63 10.1016/j.vaccine.2007.08.042 17913310 PMC2243220

[B106] Mkrtichyan M Ghochikyan A Movsesyan N Karapetyan A Begoyan G Yu J Immunostimulant adjuvant patch enhances humoral and cellular immune responses to DNA immunization DNA Cell Biol 2008 27 19 24 10.1089/dna.2007.0639 17961074 PMC2478559

[B107] de Bruijn IA Nauta J Gerez L Palache AM The virosomal influenza vaccine Invivac: immunogenicity and tolerability compared to an adjuvanted influenza vaccine (Fluad) in elderly subjects Vaccine 2006 24 6629 31 10.1016/j.vaccine.2006.05.035 16901593

[B108] Del Giudice G Hilbert AK Bugarini R Minutello A Popova O Toneatto D An MF59-adjuvanted inactivated influenza vaccine containing A/Panama/1999 (H3N2) induced broader serological protection against heterovariant influenza virus strain A/Fujian/2002 than a subunit and a split influenza vaccine Vaccine 2006 24 3063 65 10.1016/j.vaccine.2006.01.015 16464520

[B109] Kemble G Greenberg H Novel generations of influenza vaccines Vaccine 2003 21 1789 95 10.1016/S0264-410X(03)00074-4 12686096

